# Acquisition of resistance to ceftazidime-avibactam during infection treatment in *Pseudomonas aeruginosa* through D179Y mutation in one of two *bla_KPC-2_* gene copies without losing carbapenem resistance

**DOI:** 10.3389/fcimb.2022.981792

**Published:** 2022-09-02

**Authors:** Patricia García, Bárbara Brito, Manuel Alcalde-Rico, José M. Munita, Jose R. W. Martínez, Jorge Olivares-Pacheco, Valeria Quiroz, Aniela Wozniak

**Affiliations:** ^1^ Laboratory of Microbiology, Department of Clinical Laboratories, Escuela de Medicina, Pontificia Universidad Católica de Chile, Santiago, Chile; ^2^ Millennium Initiative for Collaborative Research On Bacterial Resistance (MICROB-R), Instituto de Ciencias e Innovación en Medicina (ICIM), Facultad de Medicina, Universidad del Desarrollo, Santiago, Chile; ^3^ Clinical Laboratories Network, Red de Salud UC-CHRISTUS, Santiago, Chile; ^4^ Australian Institute for Microbiology & Infection, Faculty of Science, University of Technology, Sydney, Australia; ^5^ Grupo de Resistencia Antimicrobiana en Bacterias Patógenas y Ambientales (GRABPA), Instituto de Biología, Pontificia Universidad Católica de Valparaíso, Valparaíso, Chile; ^6^ Genomics & Resistant Microbes group (GeRM), Instituto de Ciencias e Innovación en Medicina (ICIM), Facultad de Medicina Clinica Alemana, Universidad del Desarrollo, Santiago, Chile

**Keywords:** ceftazidime/avibactam resistance, *pseudomonas aeruginosa*, D179Y substitution, *bla*_KPC-2_ gene, Tn*4401b* transposon

## Abstract

Ceftazidime/Avibactam (CAZ/AVI) is frequently used to treat KPC-producing *Pseudomonas aeruginosa* (KPC-PA) and *Enterobacterales*. CAZ/AVI resistance is driven by several mechanisms. In *P. aeruginosa* this mainly occurs through alteration of AmpC, porins, and/or efflux pump overexpression, whereas in *Enterobacterales* it frequently occurs through D179Y substitution in the active site of KPC enzyme. This aminoacid change abolishes AVI binding to the KPC active site, hence inhibition is impaired. However, this substitution also decreases KPC-mediated resistance to carbapenems (“see-saw” effect). The goal of this work was to characterize the *in vivo* acquisition of CAZ/AVI resistance through D179Y substitution in a KPC-PA isolated from a hospitalized patient after CAZ/AVI treatment. Two KPC-PA isolates were obtained. The first isolate, PA-1, was obtained before CAZ/AVI treatment and was susceptible to CAZ/AVI. The second isolate, PA-2, was obtained after CAZ/AVI treatment and exhibited high-level CAZ/AVI resistance. Characterization of isolates PA-1 and PA-2 was performed through short and long-read whole genome sequencing analysis. The hybrid assembly showed that PA-1 and PA-2A had a single plasmid of 54,030 bp, named pPA-1 and pPA-2 respectively. Each plasmid harbored two copies of the *bla*_KPC_-containing Tn*4401b* transposon. However, while pPA-1 carried two copies of *bla*_KPC-2_, pPA-2 had one copy of *bla*_KPC-2_ and one copy of *bla*_KPC-33_, the allele with the D179Y substitution. Interestingly, isolate PA-2 did not exhibit the “see-saw” effect. The *bla*_KPC-33_ allele was detected only through hybrid assembly using a long-read-first approach. The present work describes a KPC-PA isolate harboring a plasmid-borne CAZ/AVI resistance mechanism based on two copies of *bla*_KPC-2_-Tn*4401b* and D179Y mutation in one of them, that is not associated with loss of resistance to carbapenems. These findings highlight the usefulness of a fine-tuned combined analysis of short and long-read data to detect similar emerging resistance mechanisms.

## Introduction

*Pseudomonas aeruginosa* is one of the most frequent causative agents of healthcare-associated infections and poses a major concern due to its outstanding ability to develop resistance to antimicrobial agents (Botelho et al., 2019). Carbapenemases are the most important acquired resistance in *P. aeruginosa* because it confers resistance to all beta-lactams and because some of them are encoded in mobile genetic elements that facilitate their dissemination. The most frequent carbapenemase in *P. aeruginosa* worldwide is VIM (Karlowsky et al., 2018; Karlowsky et al., 2019; Kiwei et al., 2021). In a recent report including more than 400 beta-lactam resistant *P. aeruginosa* isolates in Europe, 37% were VIM positive, followed by NDM and IMP (Torrens et al., 2022). In contrast to the worldwide distributed class B beta-lactamase-producing *P. aeruginosa*, KPC-producing *P. aeruginosa* (KPC-PA) has been mostly reported in American countries like Argentina (Pasterán et al., 2012; Ramírez et al., 2013), Brazil (Luna et al., 2012), Trinidad y Tobago (Akpaka et al., 2009), United States (Poirel et al., 2010), Chile (Enberg et al., 2020; Costa et al., 2021) Colombia (Villegas et al., 2007; Rada et al., 2021) and also in China (Kiwei et al., 2021). A recent Chilean study showed that among 61 carbapenemase-producing *P. aeruginosa* isolates, 54% harbored KPC, and the remaining 46% produced VIM (Costa et al., 2021).

Ceftazidime-avibactam (CAZ/AVI) is a novel antimicrobial combination that gathers ceftazidime, a well-known cephalosporin, with avibactam, a beta-lactamase inhibitor highly active against class A carbapenemases such as KPC. Notably, while avibactam also inhibits class C and some D enzymes, it does not exhibit activity against class B carbapenemases (Ehmann et al., 2012), Currently, CAZ/AVI is one of the main antibiotic alternatives used to treat infections by KPC-producing bacteria, including KPC-PA (Temkin et al., 2017) and KPC-producing *Enterobacterales* (Shields et al., 2017a). Unfortunately, CAZ/AVI resistance in *P. aeruginosa* is not uncommon, with reported resistance rates of 10-16% among isolates of carbapenem-resistant *P. aeruginosa* not harboring class B beta-lactamases (Kazmierczak et al., 2018; Karlowsky et al., 2018; Karlowsky et al., 2019). Globally, CAZ/AVI resistance occurs through several mechanisms: changes in the omega-loop of the KPC or AmpC enzymes; porin mutations; increased copy number and/or expression of *bla*_KPC_ genes; and increased expression of efflux pumps (Botelho et al., 2019; Coppi et al., 2020). D179Y substitution in the omega-loop of KPC-2 (designated KPC-33 variant) has been associated with CAZ/AVI resistance in *Enterobacterales* (Wang et al., 2020). While the specific mechanism of CAZ/AVI resistance conferred by the D179Y mutation has not been completely characterized, some studies suggest it may be related to hydrogen bonds stabilizing the interaction of the enzymatic active site with CAZ, decreasing its affinity to AVI (Winkler et al., 2015; Barnes et al., 2017). Other studies have reported that D179Y alters the acylation of KPC, decreasing the inactivation constant of AVI by ~70,000-fold (Compain and Arthur, 2017). Indeed, experiments with purified KPC-33 demonstrated a 20-fold increase in the concentration of AVI needed to inhibit 50% of CAZ hydrolysis. Also, KPC-33 exhibited a 10-fold higher efficiency hydrolyzing CAZ as compared to wild-type (WT) KPC-2 (Tsivkovski and Lomovskaya, 2020). Interestingly, the D179Y substitution restores the susceptibility to carbapenems in *Enterobacterales*, known as the “see-saw” effect (Shields et al., 2017a; Haidar et al., 2017; Shields et al., 2017b). In contrast to *Enterobacterales*, CAZ/AVI resistance in *P. aeruginosa* has been mostly associated with point mutations in chromosomal genes coding for AmpC and OXA-539 beta-lactamases (Fraile-Ribot et al., 2017; Wang et al., 2020). In addition, several changes in chromosomal genes have been reported in CAZ/AVI resistant clinical isolates of *P. aeruginosa*: *ftsI, nalD, dnaK, ctpA*, together with increased expression of MexAB-OprM efflux pump system (Castanheira et al., 2019). Herein, we report the *in vivo* emergence of high-level CAZ/AVI resistance in a clinical isolate of KPC-PA bearing two copies of *bla*_KPC_, one of which developed the D179Y substitution. Interestingly, the development of CAZ-AVI resistance did not result in concomitant carbapenem susceptibility. The finding of KPC-33 was detected through a multi-step hybrid assembly pipeline using long and short-read whole-genome sequencing (WGS) platforms.

## Materials and methods

### Patient and isolates

This work was approved by the Ethical Committee of Pontificia Universidad Católica de Chile. Isolates were obtained from a 77-year-old male patient admitted in February 2019 due to a dissecting abdominal aortic aneurysm. Two days after surgical repair, a urine culture performed due to tachycardia and abdominal pain, yielded a KPC-PA (isolate PA-1). The isolate was susceptible to amikacin, colistin and CAZ/AVI. Therapy with colistin plus CAZ/AVI was started immediately. After 16 days of therapy, fever and clinical worsening occurred and the patient developed respiratory failure and shock, requiring vasoactive support and mechanical ventilation. A culture from an endotracheal aspirate obtained at that moment yielded a CAZ/AVI resistant KPC-PA isolate (isolate PA-2). Therefore CAZ/AVI was replaced by a combination of colistin plus amikacin. The patient developed renal impairment with hypernatremia and hypokalemia that were managed with hypotonic fluids and intravenous loads, respectively. Finally, the patient exhibited a good clinical response after 48 days of colistin therapy, with disappearance of the fever and decreasing inflammatory parameters, and was discharged after 127 days of hospitalization.

### Antimicrobial susceptibility testing

Antimicrobial susceptibility was determined through the agar dilution method as per Clinical Laboratory Standards Institute (CLSI) recommendations (CLSI, 2019). Susceptibility to CAZ/AVI, ceftolozane/tazobactam and aztreonam were determined using the broth microdilution method using SensiTitre Antimicrobial Susceptibility Testing System according to manufacturer`s instructions (Thermo Fisher Scientific, United States). Susceptibility categorization was performed using the breakpoints proposed by the CLSI guidelines (CLSI, 2019).

### Carbapenemase detection

Isolates were initially assessed for carbapenemase activity through Carba-NP testing as per CLSI recommendations (CLSI, 2019). The presence of KPC, OXA-48, IMP, VIM and NDM was determined using the immunochromatographic test NG-CARBA 5 (NG-BIOTECH^®^, France). Further, molecular confirmation was performed using PCR targeting carbapenemase genes *bla*_KPC_, *bla*_OXA-48_, *bla*_NDM_, *bla*_IMP_, *bla*_IMI_, *bla*_GES_ and *bla*_VIM_, as previously described (Wozniak et al., 2012). DNA from carbapenemase-producing clinical isolates previously characterized in our laboratory was used as positive controls (Wozniak et al., 2012).

### Analysis of outer membrane proteins

SDS-PAGE analyses of the insoluble outer-membrane fraction were performed as described (Wozniak et al., 2012). Briefly, 2mL of overnight culture were centrifuged, resuspended in 10mM Tris HCl pH8, sonicated for 2min and centrifuged at 7,000g for 5min. The supernatant obtained was centrifuged at 13,000g for 45min, and the pellet was resuspended in 10mM Tris-HCl pH8 with 2% Triton X-100 and incubated at 37°C for 30min. The suspension was centrifuged at 13,000g for 45min and the pellet containing outer membrane proteins was resuspended in 100mM Tris-HCl pH8 with 2% SDS. A volume containing 50µg of protein was incubated at 98°C for 5min and analyzed in a 12.5% polyacrylamide gel at 100V. Gels were stained overnight in 0.1% Coomassie blue and washed with 1% acetic acid. Susceptible *P. aeruginosa* ATCC 27853 was included as control.

### Whole genome sequencing analysis

Isolates PA-1 and PA-2 were sequenced using both short-read and long-read WGS. For short-read sequencing, a 350 bp insert DNA library was prepared using Illumina DNA Prep Kit (formerly Nextera Flex), using the Hackflex protocol (Gaio et al., 2022). Sequencing was performed in an Illumina Platform PE150, at the University of Technology Sydney’s Bioscience Laboratory (Sydney, Australia). The Q30 obtained was > 90% for both isolates. *De novo* assembly was performed using SPADES version 3.7 package (Bankevich et al., 2012). Genomic annotation of the recovered draft genomes was performed with Prokka tool 1.11 (Seemann, 2014). Final visualization was made using Geneious™. For long-read sequencing, an Oxford Nanopore platform was used. A Ligation Library was prepared and sequenced on a GridION device at the Garvan Institute of Medical Research (Sydney, Australia).

Short-read and long-read sequences were assembled using two different hybrid assembly approaches: Unicylcler and Trycycler (Wick et al., 2017; Wick et al., 2021). Of note, Unicycler uses a short-read-first approach and Trycycler uses a long-read-first approach. For Trycycler assembly, a set of 12 assemblies made with three different assemblers, using different random subsets as input, were generated. The three assemblers used were Flye (Kolmogorov et al., 2019), Miniasm (Heng Li, 2016) and Raven (Vaser and Šikić, 2021). The clustering of assemblies was evaluated manually in a phylogenetic tree, and after confirming that the three assemblies were concordant in the plasmids and chromosomes generated, a consensus sequence was generated. The resistome characterization, genome annotation, and visualization of the hybrid assemblies were performed using the Comprehensive Antibiotic Resistance Database (CARD) Resistance Gene Identifier (RGI) v1.0.0 (McArthur et al., 2013), Prokka v1.11 (Seemann, 2014) and CGView Builder v1.0.0 (Grant and Stothard, 2008).

Raw sequence data were deposited in BioProject No. PRJNA839103 in the Sequence Read Archive of the National Center for Biotechnology Information with BioSample accession numbers SAMN28535839, SAMN28535840 for PA-1 and PA-2 respectively. Final assembly of chromosomes were deposited in Genbank with accession numbers CP097709 and CP097710, for PA-1 and PA-2 respectively. Final assembly of plasmids were deposited in Genbank with accession numbers CP097845 and CP097844 for pPA-1 and pPA-2 respectively.

### Confirmation of KPC D179Y substitution

To confirm the KPC D179Y substitution, a forward primer that included the nucleotide change G532T at the 3’ end was designed. The same primer with the WT nucleotide was also designed. The primers used were: WT-F: GCGCGCGGCGATGAGGTATC; Mut-F: GCGCGCGGCGATGAGGTATA; Rev: CTTGCCGCTCGGTGATAATC. Two PCR reactions were done for each isolate: one with primers WT-F and WT-Rev, and the other one with primers Mut-F and Mut-Rev; both PCR reactions produced a 640 bp amplicon. It was expected that PCR with primers Mut-F and Mut-Rev produced a 640 bp amplicon for PA-2, and no amplicon for PA-1. PCR with primers WT-F and WT-Rev was expected to produce a 640 bp amplicon for both isolates.

## Results

### Phenotypic and genotypic characterization of the isolates

**Figure 1:**
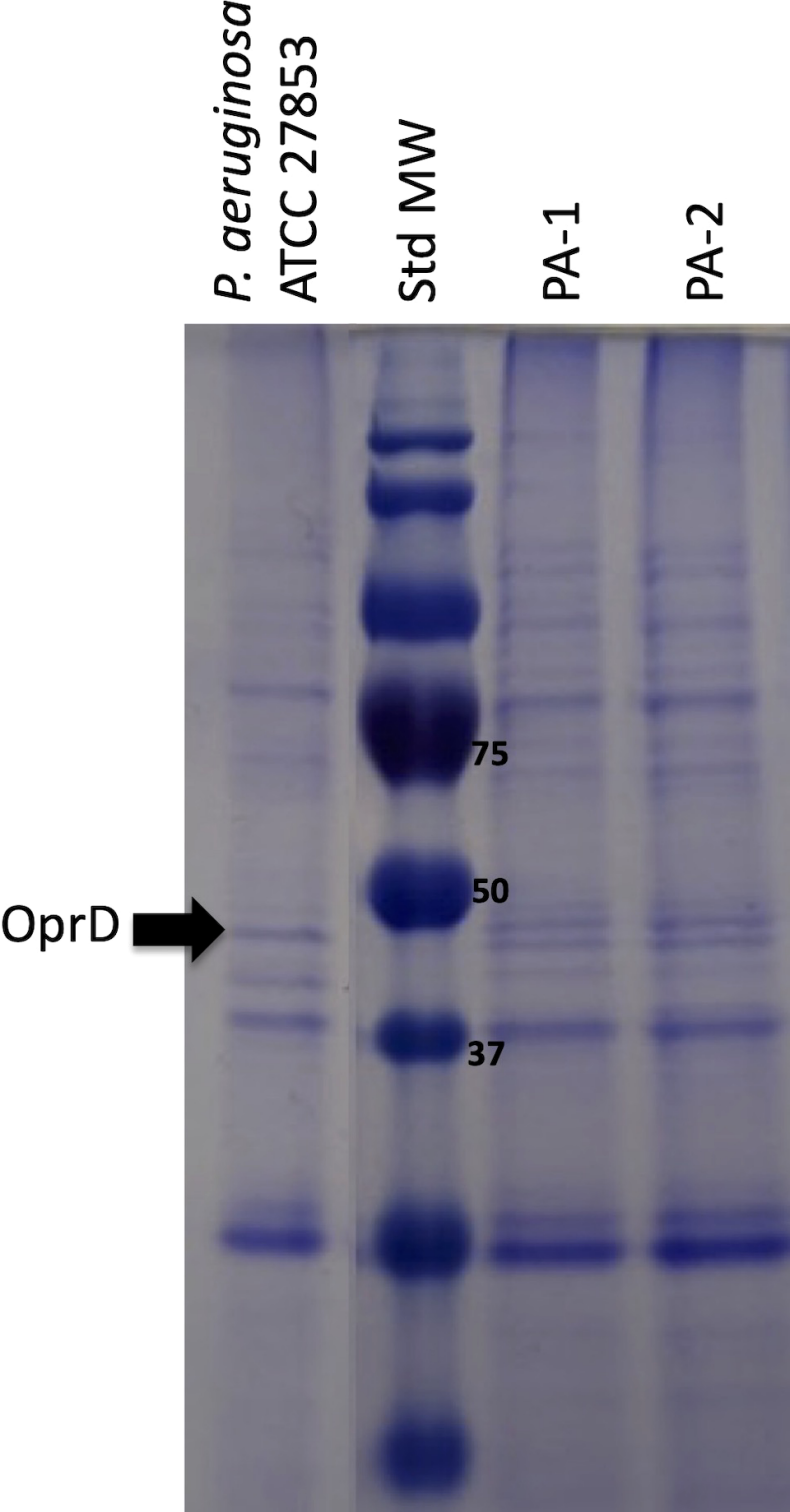
SDS-PAGE analysis of outer membrane protein extraction of isolates PA-1 and PA-2. Weight (kDa) of the Molecular Weight Standard (Std MW) bands used in protein electrophoresis is indicated next to the bands. *P. aeruginosa* ATCC 27853 was used as a control isolate. OprD was located based in its molecular weight of 48,4 kDa and according to previous reports (Rodríguez-Beltrán et al., 2015).

A summary of the susceptibility profile of PA-1 and PA-2 is presented in **Table 1**. Briefly, PA-1 exhibited resistance to all tested antimicrobials tested except for colistin, amikacin, and CAZ/AVI with minimum inhibitory concentrations (MIC) of ≤2, ≤8 and 4 μg/mL, respectively. PA-2 exhibited the same susceptibility profile of PA-1, except for CAZ/AVI, which resulted in a MIC of >256 μg/mL (**Table 1**). Both isolates exhibited carbapenemase activity as indicated by a positive Carba-NP test. Immunochromatographic detection of carbapenemases showed that both isolates produced a KPC enzyme, which was later confirmed through PCR targeting the *bla*_KPC_ gene (data not shown). Since altered production of the OprD porin may also affect CAZ/AVI activity, the porin profile was analyzed through SDS-PAGE of the insoluble outer membrane fraction. As shown in **Figure 1**, the band corresponding to OprD was absent in both clinical isolates as compared to the susceptible control. Therefore, an OprD-mediated resistance to CAZ/AVI in PA-2 was unlikely.

**Table 1:** Antimicrobial susceptibility profile of *P. aeruginosa* isolates.

	MIC (μg/mL) (category*)	
Antimicrobial Agent	PA-1	PA-2
amikacin	≤8 (S)	≤8 (S)
gentamycin	8 (I)	8 (I)
ampicillin	>32 (R)	>32 (R)
ampicillin/sulbactam	>32 (R)	>32 (R)
cefoperazone/sulbactam	>64 (R)	>64 (R)
piperacillin/tazobactam	>128 (R)	>128 (R)
cefixime	>4 (R)	>4 (R)
cefepime	>32 (R)	>32 (R)
cefotaxime	>128 (R)	>128 (R)
ceftazidime	>128 (R)	>128 (R)
**ceftazidime/avibactam^#^**	**4 (S)**	**>256 (R)**
ceftolozane/tazobactam^#^	>32 (R)	>32 (R)
aztreonam^#^	>32 (R)	>32 (R)
cotrimoxazole	>80 (R)	>80 (R)
meropenem	>16 (R)	>16 (R)
imipenem	>16 (R)	>16 (R)
colistin	≤2 (S)	≤2 (S)
nitrofurantoin	>128 (R)	>128 (R)
fosfomycin	>256 (R)	>256 (R)
ciprofloxacin	>4 (R)	>4 (R)

*Categories were assigned according to CLSI 2019 guidelines: R, resistant; I, intermediately resistant; S, susceptible. MIC, Minimum Inhibitory Concentration. ^#^Antimicrobials that were evaluated through broth microdilution method (Sensititre, Thermo Scientific).

*Susceptibility pattern of PA-1 and PA-2 differ only in ceftazidime/avibactam MIC (bold values).

### Short-read and long-read WGS analyses

Our genomic *in-silico* MLST analysis revealed both isolates belonged to ST654 and they harbored a *bla*_KPC-2_ gene embedded in a typical Tn*4401b* transposon. Mutations in several chromosomally-encoded genes frequently associated with CAZ/AVI resistance were analyzed^;^ no differences were found between both isolates. However, several mutations in genes associated with CAZ/AVI resistance were present in both isolates respect to WT strain PAO-1 (**Table 2**). An 8 bp deletion at nucleotide 235 was observed in *mexT*, which resulted in a frameshift from amino acid 80. In addition, *mexZ* had a 2 bp deletion at positions 550 and 551, which produced a frameshift from amino acid 184. Both changes in MexT and MexZ were predicted to result in a loss of protein function according to PROVEAN analysis (Choi et al., 2012). Other changes observed in chromosomal genes were predicted to produce minor or no changes in protein function according to PROVEAN analysis (**Table 2**). Both PA-1 and PA-2 had the same allele of the endogenous *ampC* gene (PDC-3), WT alleles of *mexAB-oprM* operon, the same polymorphisms in transcriptional regulators MexT, MexD, MexZ, NalC and NalD, and the same variant of OprD porin. Despite OprD porin had a deletion of two amino acids and 29 amino acid substitutions respect to PAO-1 strain, these alterations were neutral according to PROVEAN analysis. This OprD protein variant was reported as functional (Suresh et al., 2020). According to these data, no evident differences were found between both isolates that could explain CAZ/AVI resistance in PA-2.

**Table 2:** Sequence analysis of PA-1 and PA-2 acquired resistance genes and chromosomal genes associated with CAZ/AVI resistance compared to PAO-1 control strain.

	PA-1	PA-2	PROVEAN*
**Acquired resistance genes**	*bla_KPC-2_*	*bla_KPC-2_, bla_KPC-33_*	NA
**Chromosomal genes associated to CAZ/AVI resistance^&^**			
***ampC***	T105A (PDC-3 alelle)	T105A (PDC-3 alelle)	Neutral
***bla*_OXA-397_**	WT	WT	NA
***mexAB-oprM***	WT	WT	NA
***oprD***	V127L, E185Q, P186G, V189T, E202Q, I210A, E230K, S240T, N262T, T276A, A281G, K296Q, Q301E, R310E, G312R, A315G, L347M, M372V, S373D, D374S, N375S, N376S, V377S, K380Y, N381A, Y382G, G383L, S403A, Q424E, Δ378G, Δ379Y	V127L, E185Q, P186G, V189T, E202Q, I210A, E230K, S240T, N262T, T276A, A281G, K296Q, Q301E, R310E, G312R, A315G, L347M, M372V, S373D, D374S, N375S, N376S, V377S, K380Y, N381A, Y382G, G383L, S403A, Q424E, Δ378G, Δ379Y	Neutral
***nalD***	T43I	T43I	Neutral
***nalC***	G71E	G71E	Neutral
***mexZ***	550C and Δ551T: Frameshift from aminoacid 184	Δ550C and Δ551T: Frameshift from aminoacid 184	Deleterious
***mexD***	E257Q, S845A	E257Q, S845A	Neutral
***mexT***	Δ235-241 (8 bp deletion): Frameshift from aminoacid 80	Δ235-241 (8 bp deletion): Frameshift from aminoacid 80	Deleterious
***mexR***	WT	WT	NA
***dnaK***	WT	WT	NA
***ftsI***	WT	WT	NA
***nfxB***	WT	WT	NA
***esrC***	WT	WT	NA
***ctpA***	WT	WT	NA

^&^, All the amino acid changes are expressed based in PAO-1 sequence.

*, Predicted impact of amino acid change on protein function.

NA, Not applicable; WT, Wild Type.

**Figure 2:**
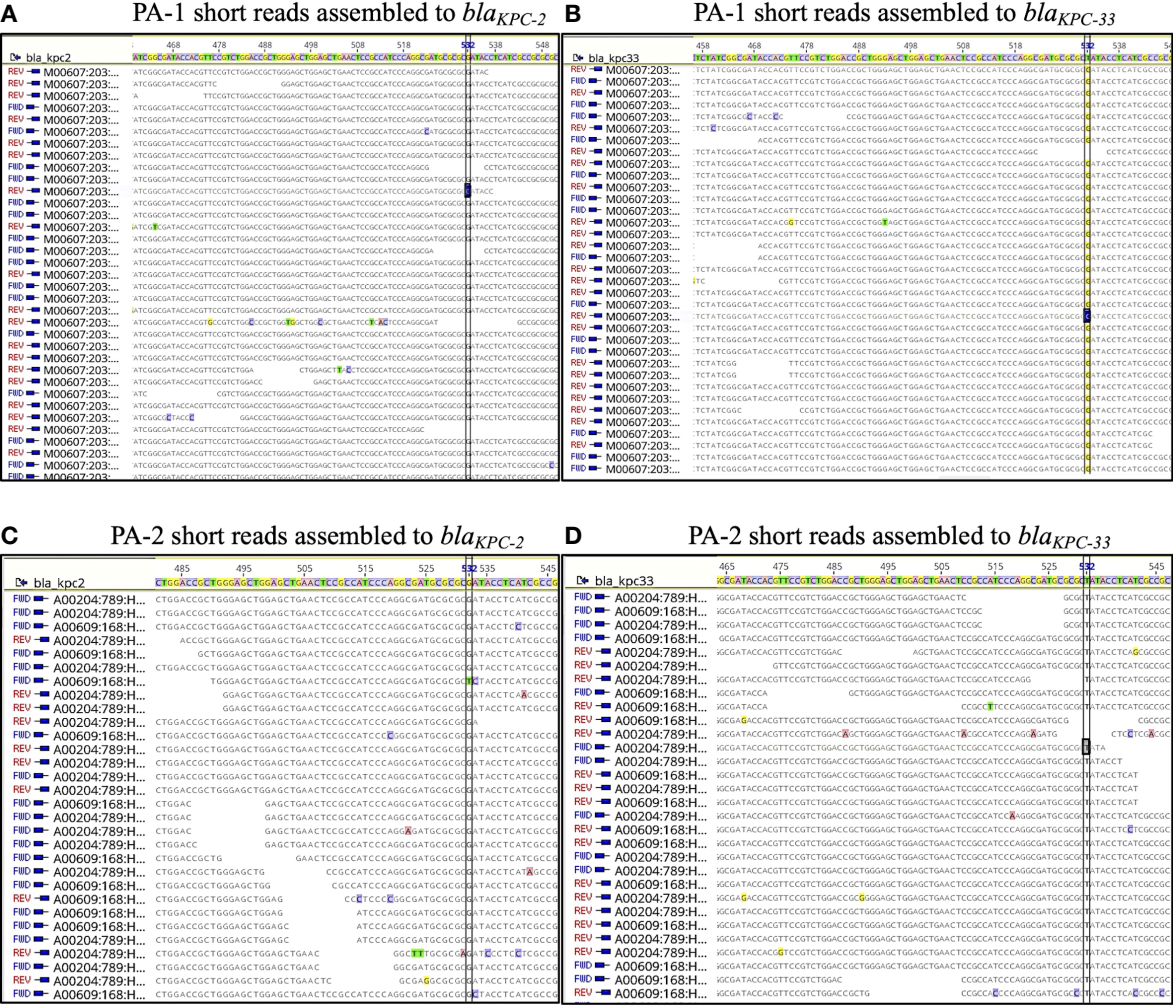
Short reads of PA-1 **(A, B)** and PA-2 **(C, D)** mapped against *bla*_KPC-2_ **(A, C)** and *bla*_KPC-33_ sequences **(B, D)**. Position 532 is indicated in a vertical rectangle, and it has “G” in *bla*_KPC-2_ and “A” in *bla*_KPC-33._.

**Figure 3:**
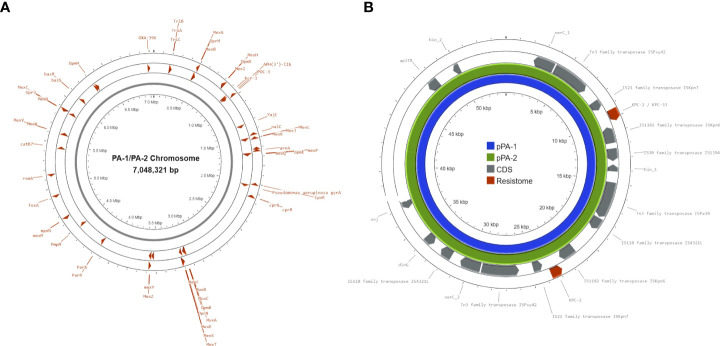
Chromosomes of isolates PA-1 **(A)** and PA-2 **(B)** and plasmids pPA-1 and pPA-2 C. Red arrowheads in A and B represent genes that are associated with antimicrobial resistance. The resistome of plasmids pPA-1 and pPA-2 C is composed only by two *bla*_KPC-2_ genes in pPA-1 and both *bla*_KPC-2_ and *bla*_KPC-33_ genes in pPA-2. Grey bars represent coding sequences (CDS) whose gene names are shown for annotated genes or not shown for hypothetical proteins (hp). Transposase genes: associated with transposition events; *hin_1, hin_2, dinG, soj:* genes associated with DNA processing and repair; *xerC:* gene associated with plasmid replication control.

To obtain a more detailed genomic analysis, hybrid assemblies were performed using short and long-read data for each isolate. Using Unicycler (short-read-first approach), isolate PA-1 generated two circular scaffolds of 7,046,950 bp and 54,030 bp long, and isolate PA-2 generated two linear scaffolds of 6,969,342 bp and 71,243 bp, and a circular scaffold of 40,443 bp. Scaffolds of 54,030 bp (PA-1) and 40,443 bp (PA-2) corresponded to plasmids based on the presence of plasmid replication control genes and were named pPA-1 and pPA-2, respectively. Plasmid pPA-1 harbored two copies of the *bla*_KPC_ gene, each one contained in a Tn*4401b* transposon separated by 8,687 bp, whereas plasmid pPA-2 had only one copy of the Tn*4401b* transposon containing *bla*_KPC-2_ according to Unicycler assembly. In contrast, using Trycycler (long-read-first approach) both isolates generated the same two circular scaffolds of 7,048,060 bp and 54,030 bp long. The Trycycler assembly showed both plasmids, pPA-1 and pPA-2 carried two copies of *bla*_KPC_, both of which were harbored in a Tn4401b transposon structure. Interestingly, while both copies in PA-1 were WT *bla*_KPC-2_, in isolate PA-2 one of the copies was a WT *bla*_KPC-2_ gene and the other copy had a G532T base change that produced D179Y substitution in the omega-loop of KPC-2 and was therefore a *bla*_KPC-33_ allele. To further corroborate this point mutation, the short reads of PA-1 and PA-2 were mapped against both *bla*_KPC_ alleles. No reads of PA-1 aligned with the *bla*_KPC-33_ sequence. In contrast, analysis of PA-2 showed a balanced mapping of reads aligning with *bla*_KPC-2_ and *bla*_KPC-33_, respectively (**Figure 2**). A pair-wise alignment between the chromosomes of PA-1 and PA-2 demonstrated they were identical except for four 1-bp insertions/deletions and five 1-bp substitutions (99.99% identity) (**Figure 3**). Pair-wise alignment of plasmids pPA-1 and pPA-2 showed they were identical, except for nucleotide change in position G532T of the *bla*_KPC_ gene, which resulted in the D179Y amino acid substitution (**Figure 3**).

### Confirmation of the co-existence of *bla*_KPC-2_ and *bla*_KPC-33_ alleles in PA-2 by PCR

**Figure 4:**
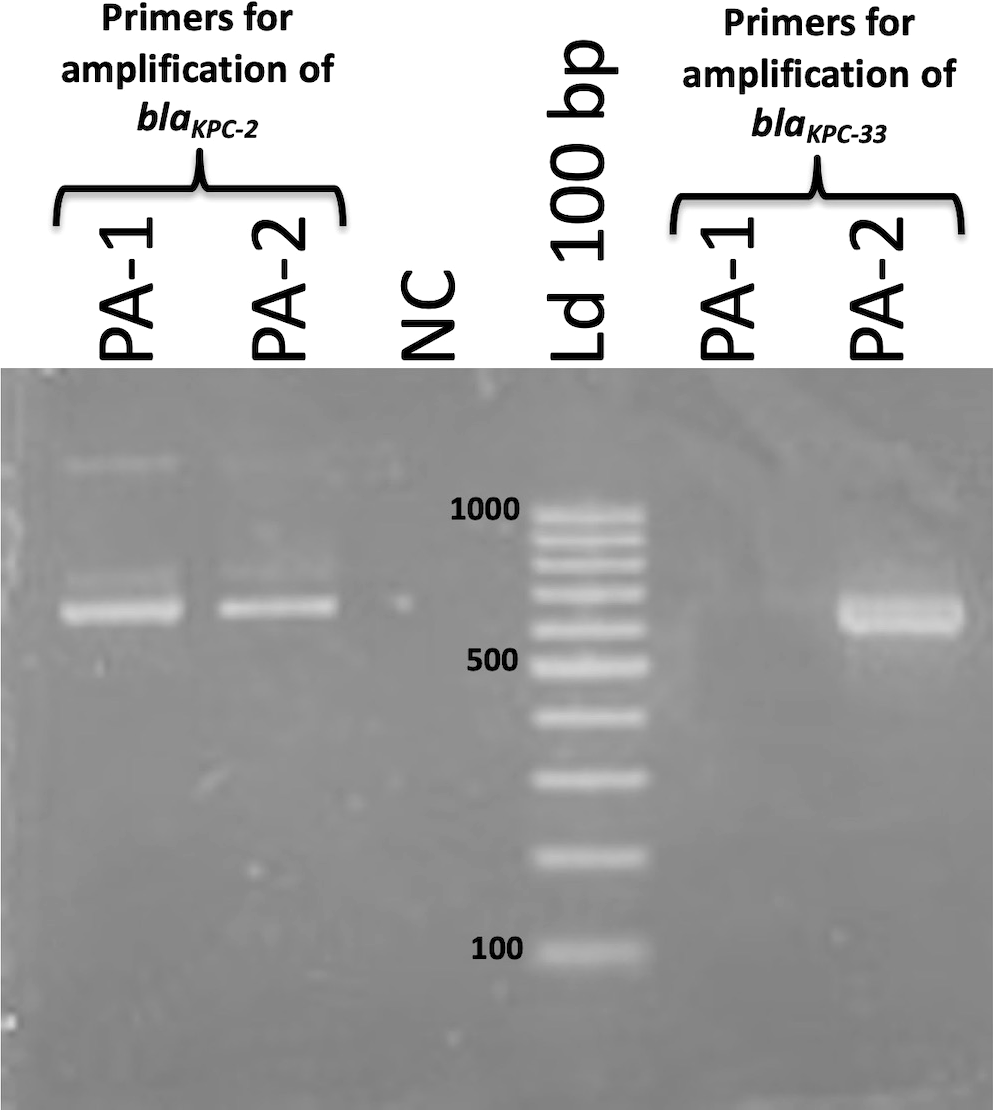
Agarose gel electrophoresis of PCR products obtained in the amplification of PA-1 and PA-2 DNA using primers for amplification of *bla*_KPC-2_ (left) and *bla*_KPC-33_ (right). Both primer pairs amplify a fragment of 640 bp. Band sizes (bp) of the Molecular Weight Standard (Ld 100 bp) are indicated next to the bands.

The single nucleotide substitution of *bla*_KPC-33_ with respect *bla*_KPC-2_ was further confirmed by PCR using primers specific to each of the alleles. To do so, we designed two identical forward primers with a single nucleotide difference between them in the 3`end, which corresponded to the G532T change associated to the *bla*_KPC-33_ allele. This nucleotide variation prevented the complete 3`end annealing of the primer against WT *bla*_KPC-2_. PCR with primers directed to *bla*_KPC-2_ amplified a 640 bp fragment as expected in both isolates PA-1 and PA-2, while primers directed to *bla*_KPC-33_ only amplified the 640 bp fragment in PA-2 (**Figure 4**). Therefore, the presence of *bla*_KPC-33_ was confirmed only in isolate PA-2.

## Discussion

In the present work, we characterized a KPC-PA clinical isolate that developed CAZ/AVI resistance *in vivo* through D179Y mutation in one of two *bla*_KPC-2_ genes that were harbored in a plasmid. Isolate PA-2 had >64-fold increase in CAZ/AVI MIC with respect to PA-1, but did not exhibit a decrease in carbapenem resistance (“see-saw” effect) as observed in *Enterobacterales* (Shields et al., 2017a; Shields et al., 2017b; Tsivkovski and Lomovskaya, 2020). The presence of both KPC-2 and KPC-33 is most likely responsible for the absence of the “see-saw” effect. This “all in one” resistant phenotype includes resistance to CAZ/AVI and to carbapenems, since the cost associated with the acquisition of *bla*_KPC-33_ alone is offset by *bla*_KPC-2_. In a previous work from our group, we described a D179Y mutation in the *bla*_KPC-2_ gene of a clinical isolate of *P. aeruginosa*, but CAZ/AVI resistance was not addressed that time (Wozniak et al., 2019). This KPC-PA isolate had a negative immunochromatographic test, therefore, it is unlikely that it had an additional WT *bla*_KPC-2_ copy. However, this isolate was resistant to meropenem and imipenem, therefore no “see-saw” effect was observed. According to these findings, the “see-saw” effect may be observable in *Enterobacterales*, but not necessarily in *P. aeruginosa*. This could be explained because *Enterobacterales* are much more dependent on carbapenemase function than *P. aeruginosa*, which has a variety of additional mechanisms for carbapenem resistance, e.g., efflux pump overexpression, porin alteration, AmpC and OXA enzymes alterations, among others (Botelho et al., 2019). In fact, several mutations in chromosomal genes associated with CAZ/AVI resistance were observed in both PA-1 and PA-2. Notably, isolate PA-1 was susceptible to CAZ/AVI, despite having these alterations. Most of the mutations observed have been previously reported in CAZ/AVI resistant isolates. NalC is a repressor of the positive regulator of MexAB-OprM and G71E mutation was described in aztreonam resistant isolates (Braz et al., 2016). NalD is a repressor of MexAB-OprM operon, and the mutation observed in this work has been observed in CAZ/AVI resistant *P. aeruginosa* (Castanheira et al., 2019). MexT is a transcriptional regulator that activates the expression of MexEF-OprN multidrug efflux system. The mutation observed in MexT has been reported in multi-drug resistant *P. aeruginosa* with decreased expression of OprD porin (Epp et al., 2001). MexZ is a repressor of the MexXY operon and alterations in *mexZ* have been described in CAZ/AVI resistant *P. aeruginosa* isolates (Castanheira et al., 2019). Mutation T105A in AmpC (PDC-3 allele) confers reduced susceptibility to imipenem, ceftazidime, and cefepime (Rodríguez-Martínez et al., 2009). Our results suggest that these mutations may not confer CAZ/AVI resistance alone, but they may contribute to overall resistance.

Hybrid assembly using short and long-read data is an excellent approach to predict putative plasmids, resistance genes, and beta-lactamase gene variants (Khezri et al., 2021). The G532T mutation in *bla*_KPC_ gene responsible for D179Y substitution, was observable only in the hybrid assembly obtained with Trycycler; it was not observable in the short-read assembly, nor in the hybrid assembly made with Unicycler. The short reads (350 bp long) are unable to cover both KPC-Tn4401 copies (28,699 bp) present in plasmids pPA-1 and pPA-2, and Unicycler considered both *bla*_KPC-2_ and *bla*_KPC-33_ alleles as a single gene. In contrast, long reads (with an average length of 40,000 bp) can cover both KPC-Tn4401 copies. However, Trycycler requires manual intervention from the user, it is more time-consuming and therefore is more challenging for high-throughput assembly. In a previous report a short-read WGS analysis of a CAZ/AVI resistant clinical isolate of KPC-producing *K. pneumoniae* determined that 46% of *bla*_KPC_ reads were *bla*_KPC-33_ and 54% were *bla*_KPC-2_ (Gaibani et al., 2020). A similar short-read WGS analysis of a clinical isolate of *K. pneumoniae* that developed high-level CAZ/AVI resistance after antibiotic treatment showed that 28% of the reads covering *bla*_KPC_ gene were *bla*_KPC-2_ and 72% were *bla*_KPC-33_ (Sun et al., 2021). Both articles mentioned above concluded that these isolates consisted of a mixed population containing both CAZ/AVI-resistant and CAZ-AVI-susceptible bacteria, which is commonly interpreted as heteroresistance. Our results show that interpretation of short-read data must be done with caution because this strategy not always allows discrimination between mixed populations and gene duplication with subsequent mutation. These findings underscore the actual value of long-read WGS methods and hybrid assembly combining short and long-read data.

The plasmids described in this work, pPA-1 and pPA-2, were similar to pPA2047 a 43,660 bp plasmid from *P. aeruginosa* recently reported in Argentina (>65% identity) (Cejas et al., 2022), and to pPae-13, a plasmid that was recently reported in a KPC-PA clinical isolate in the same Institutional Hospital as the present work (>60% identity) (Wozniak et al., 2021). It is possible that replicative transposition of *bla*_KPC_-containing Tn*4401b* transposon in pPae-13 plasmid had produced pPA-1, and pPA-2 subsequently evolved through point mutation.

Development of high-level CAZ/AVI resistance upon CAZ/VI treatment greatly compromises the usefulness of this antibiotic combination for the treatment of KPC-PA infections, being colistin the most plausible option, with the subsequent associated renal impairment, like the one described in the present case. The lack of better therapeutic options together with the genetic characteristics of KPC-PA, i.e., location of *bla_KPC_* genes in Tn*4401b* transposon, carriage by the high-risk clone ST654, make this finding highly concerning, particularly in regions where KPC-PA is frequent.

## Data availability statement

The datasets presented in this study can be found in online repositories. The names of the repository/repositories and accession number(s) can be found below: https://www.ncbi.nlm.nih.gov/genbank/, CP097709 https://www.ncbi.nlm.nih.gov/genbank/, CP097710 https://www.ncbi.nlm.nih.gov/, SAMN28535839 https://www.ncbi.nlm.nih.gov/, SAMN28535840.

## Ethics statement

The information about human patients were reviewed and approved by the Institutional Review Board of the Ethics and Biosafety Committee of Pontificia Universidad Católica de Chile.

## Author contributions

AW, PG and BB conceived the idea and designed the experiments. JO-P, MA-R and JMu supervised the work. VQ, AW and BB performed the experiments in the study. BB, JMa, MA-R and AW performed bioinformatic analysis. PG, AW, BB, and MA-R wrote the paper. All authors read and approved the final manuscript.

## Funding

This work was supported by research funds from SENTRY (Antimicrobial Resistance Surveillance Program), the Red de Salud UC-Christus and the Department of Clinical Laboratories at the School of Medicine of Pontificia Universidad Católica de Chile. Funding for whole-genome sequencing was obtained through the Key Technology Partnership Program from the University of Technology Sydney. This work was partially funded by the Agencia Nacional de Investigation y Desarrollo (FONDECYT regular award #1211947; JMu), and the Millennium Science Initiative, Millennium Initiative for Collaborative Research on Bacterial Resistance, Government of Chile (award number NCN17_081; JMM)

## Acknowledgments

We thank the staff of the Microbiology Laboratory of the Red de Salud UC-CHRISTUS for their help in the technical aspects of this work. We also thank the Millennium Science Initiative of the Ministry of Economy, Development and Tourism, Government of Chile. We would like to express our gratefulness to the “*Pseudomonas* group”, a collaborative team including researchers from various Chilean Universities: Pontificia Universidad Católica de Chile, Pontificia Universidad Católica de Valparaíso, Universidad de Concepción; Universidad del Desarrollo.

## Conflict of interest

The authors declare that the research was conducted in the absence of any commercial or financial relationships that could be construed as a potential conflict of interest.

The handling editor AO-C declared a past collaboration with the authors AW, PG, MA-R, JM, JO-P.

## Publisher’s note

All claims expressed in this article are solely those of the authors and do not necessarily represent those of their affiliated organizations, or those of the publisher, the editors and the reviewers. Any product that may be evaluated in this article, or claim that may be made by its manufacturer, is not guaranteed or endorsed by the publisher.
